# Low prevalence of transmitted K65R and other tenofovir resistance mutations across different HIV-1 subtypes: implications for pre-exposure prophylaxis

**DOI:** 10.7448/IAS.15.2.17701

**Published:** 2012-10-15

**Authors:** Philip A Chan, Austin Huang, Rami Kantor

**Affiliations:** Division of Infectious Diseases, The Warren Alpert Medical School of Brown University, Providence, Rhode Island, United States

**Keywords:** HIV, drug resistance, PrEP, tenofovir, K65R, non-B subtypes

## Abstract

**Introduction:**

Tenofovir-containing regimens have demonstrated potential efficacy as pre-exposure prophylaxis (PrEP) in preventing HIV-1 infection. Transmitted drug resistance mutations associated with tenofovir, specifically the reverse transcriptase (RT) mutation K65R, may impact the effectiveness of PrEP. The worldwide prevalence of transmitted tenofovir resistance in different HIV-1 subtypes is unknown.

**Methods:**

Sequences from treatment-naïve studies and databases were aggregated and analyzed by Stanford Database tools and as per the International AIDS Society (IAS-USA) resistance criteria. RT sequences were collected from GenBank, the Stanford HIV Sequence Database and the Los Alamos HIV Sequence Database. Sequences underwent rigorous quality control measures. Tenofovir-associated resistance mutations included K65R, K70E, T69-insertion and ≥3 thymidine analogue mutations (TAMs), inclusive of M41L or L210W.

**Results:**

A total of 19,823 sequences were evaluated across diverse HIV-1 subtypes (Subtype A: 1549 sequences, B: 9783, C: 3198, D: 483, F: 372, G: 594, H: 41, J: 69, K: 239, CRF01_AE: 1797 and CRF02_AG: 1698). Overall, tenofovir resistance prevalence was 0.4% (n=77/19,823, 95% confidence interval or CI: 0.3 to 0.5). K65R was found in 20 sequences (0.1%, 95% CI: 0.06 to 0.15). Differences in the prevalence of K65R between HIV-1 subtypes were not statistically significant. K70E and ≥3 TAMs were found in 0.015% (95% CI: 0.004 to 0.04) and 0.27% (95% CI: 0.2 to 0.4) of sequences, respectively.

**Conclusions:**

Prevalence of transmitted K65R and other tenofovir resistance mutations across diverse HIV-1 subtypes and recombinants is low, suggesting minimal effect on tenofovir-containing PrEP regimens.

## Introduction

Novel biomedical interventions have gained widespread attention as possible strategies to prevent new HIV-1 infections. Among these, use of tenofovir-containing regimens as pre-exposure prophylaxis (PrEP) have demonstrated efficacy as both oral and topical microbicide preparations in several recent trials [1,2]. Tenofovir disoproxil fumarate (TDF) is a nucleotide reverse transcriptase inhibitor (NRTI) that is rapidly absorbed and obtains prolonged concentrations in serum and genital tissues [3], which are advantageous qualities for a prophylactic drug. In the landmark PrEP trials, individuals who acquired HIV while on PrEP did not demonstrate resistance to tenofovir, which may be explained by tenofovir's high genetic barrier for resistance [1,2]. However, the efficacy of prevention of HIV transmission from infected to uninfected individuals using tenofovir-containing PrEP regimens may be limited by the existence of viruses harbouring tenofovir-associated resistance mutations in the transmitting individual.

Current HIV treatment guidelines in developed countries recommend obtaining resistance testing at diagnosis and before initiation of anti-retrovirals to evaluate for transmitted drug resistance [4]. In people living with HIV, using anti-retrovirals to which HIV is resistant results in progression of disease, including AIDS and death [5]. In HIV-uninfected individuals, transmitted drug resistance may cause anti-retrovirals used for PrEP to have decreased or no preventative activity.

There are several mutations in the HIV-1 reverse transcriptase (RT) gene that confer resistance to tenofovir, though different expert mutation lists may differ in their characteristics and individual weight. A lysine to arginine mutation at position 65 of the RT (K65R), which confers a two- to four-fold decrease in susceptibility to tenofovir [6], is included on all drug resistance lists and has historically been considered the classic tenofovir-associated mutation. This mutation is evident in individuals who have failed tenofovir-containing regimens [7] and also results in resistance to abacavir, didanosine and stavudine [8]. The reported prevalence of transmitted K65R is low. Regional surveys of its transmission demonstrate low prevalence (<1 to 3%) mostly in the United States and Europe where tenofovir is widely used [9]. These reports are mainly from patients living with HIV-1 subtype B, the predominant subtype in North America, Europe and Australia. However, HIV-1 non-B subtypes account for 90% of the worldwide infections with subtype C constituting the majority [9].

Sequence diversity in different HIV-1 subtypes may influence the emergence of drug resistance mutations. K65R may be more likely to develop in subtype C, due to the nucleic acid variation surrounding this codon [10,11]. The overall impact of HIV-1 genetic diversity on transmission of resistance is unclear.

As PrEP is incorporated into HIV clinical care worldwide, the global prevalence of tenofovir resistance mutations in HIV-1-circulating variants is an important factor. We determined the prevalence of K65R and other mutations associated with tenofovir resistance by direct examination of RT sequences from treatment-naïve individuals across different HIV-1 subtypes and recombinant forms.

## Methods

We reviewed studies evaluating drug resistance in HIV-1 positive, anti-retroviral treatment-naïve individuals reported in PubMed (ncbi.nlm.nih.gov/pubmed) through 31 July 2010. For each study with available data, RT sequences were obtained from GenBank (ncbi.nlm.nih.gov/genbank), Stanford HIV Sequence Database (hivdb.stanford.edu) and/or Los Alamos HIV Databases (hiv.lanl.gov). All databases were last accessed on 31 July 2010. Additional data obtained included country of study and year when the sample was collected, if available. The timing of sequences relative to HIV seroconversion was unknown. HIV-1 subtypes (A, B, C, D, F, G, H, J, K, CRF01_AE and CRF02_AG) were determined by the Stanford HIV Database. Sequences underwent rigorous quality control measures, including inspection for duplications, frameshifts, insertions, deletions, stop codons and genetic distances using SQUAT methods [12]. A sequence was excluded from further analysis based on quality control criteria, (1) if it was a duplicate of another sequence in the dataset; (2) if there was more than one stop codon present; in cases of one stop codon, none occurred at a tenofovir-resistance-associated position; (3) if there were more than 29 ambiguous amino acids in the RT sequence, based on the highest SQUAT cut-offs; and (4) if the sequence did not extend from RT position 41 to 219 (the region associated with tenofovir resistance positions). Resistance mutations and susceptibility prediction were identified according to Stanford HIV Sequence Database tools (hivdb.stanford.edu) and interpreted based on the 2011 IAS-USA mutation list [4]. Mixtures of resistance and non-resistance viral populations were considered resistant. Tenofovir-associated resistance mutations included K65R, T69 insertion, K70E and ≥3 thymidine-analogue mutations (TAMs; M41L, D67N, K70R, L210W, T215F/Y, K219Q/E), inclusive of either M41L or L210W. Mutation proportions were compared among subtypes using Fisher's exact test.

## Results

Two hundred and three studies were reviewed and 23,291 RT sequences of diverse HIV-1 subtypes and recombinant forms from across the world were collected (Table 1). Quality control measures resulted in the removal of 2083 duplicate sequences, 48 sequences with more than one stop codon, 156 sequences with greater than 29 ambiguous amino acids, 7 sequences which were group O, 265 sequences that did not start at position 41 and 909 sequences that did not extend to position 219. The final analyzed dataset included 19,823 sequences of subtypes A (n=1549), B (n=9783), C (n=3198), D (n=483), F (n=372), G (n=594), H (n=41), J (n=69), K (n=239), CRF01_AE (n=1797) and CRF02_AG (n=1698).

**Table 1:** Characteristics of 19,823 sequences from HIV-positive anti-retroviral-naïve individuals

	Characteristic	Number of sequences
Country	Africa	4686
	Asia	4951
	Europe	5376
	North America	2221
	Central America	410
	South America	2186
Subtype	A	1549
	B	9783
	C	3198
	D	483
	F	372
	G	594
	H	41
	J	69
	K	239
	CRF01_AE	1797
	CRF02_AG	1698
Year of sequence	<2000	3486
	2000 to 2004	10,980
	2005 to 2008	5364
Database	GenBank	554
	Los Alamos	17
	Stanford	19,259

There were 20/19,823 sequences with K65R (0.1%, 95% CI: 0.06 to 0.15; Table 2), 14/9783 (0.14%) in subtype B from North/Central America and Europe, 3/3198 (0.09%) in subtype C from Africa, 2/1797 (0.11%) in CRF01_AE from Vietnam and 1/594 (0.17%) in subtype G from Europe. There was a trend in K65R occurring more frequently in subtype B (14/9783, 0.14%) compared to aggregated non-subtype B sequences (6/10,034, 0.06%; *p*=0.07). There was no significant difference in K65R occurrence in subtype C (3/3198, 0.09%) compared to aggregated non-subtype C sequences (17/16,608, 0.10%; *p*=1) or to subtype B sequences (*p*=0.80). Of the 20 K65R-containing sequences, seven had no additional resistance mutations, two had one mutation, four had two mutations, five had three mutations and two sequences had four or more mutations. Two of the sequences had additional NRTI-associated mutations, two had additional NNRTI-associated mutations and nine sequences had additional mutations in both classes.

**Table 2:** Characteristics of K65R-containing sequences from anti-retroviral-naïve HIV-positive individuals

Region	Country	Accession or patient ID*	Subtype	Year	NRTI	NNRTI
Africa	Senegal	AJ583739*	C	1999	None	V108I
	South Africa	DQ445633*	C	1999	None	None
	South Africa	AY901973*	C	1999	None	None
Asia	Vietnam	AB519458*	CRF01_AE	2008	Q151M, M184V	Y181C
	Vietnam	AB519470*	CRF01_AE	2008	Q151M, M184V	Y181C
	Taiwan	EU164857*	B	2001	None	None
	Taiwan	EU164897*	B	2004	M184IM	K103KNRS, V106IL
	Taiwan	EU164893*	B	2004	None	None
Europe	Portugal	AF504588*	B	2000	None	None
	Spain	AF479608*	B	2001	None	K103N, Y181C, G190A
	Germany	GQ400763*	B	2003	M184V, T215F	None
	Serbia	GQ399262*	G	2003	Q151M	Y181C
Central America	Honduras	EU312786*	B	2003	M184V	K103N, Y181C
North America	United States	CR2**	B	2007	K219KQ	K103N
	United States	CR32**	B	2007	A62V, M184V	K103N, Y181C
	United States	CR79**	B	2005	M184V	None
	United States	CR143**	B	2006	None	None
	United States	CR158**	B	2006	L74LV, Y115FY	L100I, K103N, H221HY
	United States	CR181**	B	2006	None	None
	United States	CR244**	B	2006	M184V	K101E

ID=identification

* Genbank ID

** Stanford HIV Sequence Database ID

Transmitted K65R was observed among sequences that were collected from 1999 until 2008. There were three sequences from 1999, one from 2000, two from 2001, three from 2003, two from 2004, one from 2005, four from 2006, two from 2007 and two from 2008. There was no significant difference between the number of K65R sequences that were collected in the five-year period between 1999 and 2003 (when tenofovir became more widely used) and those collected in the five-year period between 2004 and 2008 (9/20 vs. 11/20; *p*=0.80).

In addition to K65R, other transmitted tenofovir-associated resistance mutations included K70E, seen in three subtype B sequences (0.015%, 95% CI: 0.004 to 0.04) and three or more TAMs (inclusive of either M41L or L210W) seen in 54/19,823 sequences (0.27%, 95% CI: 0.2 to 0.4), the majority of which (46/54) were subtype B sequences. There were no occurrences of T69 insertions. The overall prevalence of any tenofovir-associated mutation in this sequence dataset was 0.4% (n=77/19,823, 95% CI: 0.3 to 0.5).

All 20 sequences with K65R had intermediate or high-level predicted resistance to tenofovir and the three sequences with K70E had low-level predicted tenofovir resistance. Of the 54 sequences with three or more TAMs, 24 (44%) demonstrated high level predicted tenofovir resistance and 30 (56%) demonstrated intermediate predicted resistance.

## Discussion

In this analysis of globally available HIV-1 *pol* sequences, the prevalence of RT mutations associated with tenofovir resistance, including K65R, across diverse subtypes and recombinant forms is low (0.10%, 20/19,823). These data, derived from direct sequence analysis and not reported results, suggest that at the current time, transmitted resistance on a global scale may have a minimal effect on tenofovir-based PrEP effectiveness.

The previously reported transmission of K65R in subtype B infected patients is less than 1%, reflecting resource-rich settings where tenofovir and other NRTIs are widespread. In two large surveillance studies from the United States, out of 2030 newly HIV-positive individuals and 1585 treatment-naïve individuals, there were only one (0.9%) and three (0.19%) occurrences of K65R, respectively [13,14]. Our results support the low prevalence of K65R and extend the finding to other, globally predominant, non-B subtypes. Subtype C, the predominant worldwide subtype, did not have an increased rate of K65R in this dataset, despite previously reported higher rates of occurrences of this mutation in this subtype [10]. The observed non-significant trend in K65R prevalence in subtype B compared to non-subtype B sequences likely reflects higher reporting from, and the widespread use of, tenofovir-containing regimens in resource-rich settings, where subtype B is predominant. There was no detectable difference in the occurrence of K65R before or after 2004, despite the increased use of tenofovir as part of first-line or advanced anti-retroviral regimens since 2004. The limited numbers of available naïve sequences of each of the non-B subtypes, including subtype C, and the rare occurrence of K65R overall, mandate further monitoring to examine K65R global occurrence and transmission.

The most common non-K65R tenofovir-associated resistance mutations were TAMs, which are common in patients failing NRTI-based regimens. Though these mutations may be transmitted more commonly as individual mutations, sequences with three or more TAMs (inclusive of either M41L or L210W) can confer significantly reduced tenofovir activity and were observed in our dataset. Other tenofovir-associated mutations, such as K70E and T69 insertions, were either rare or absent. These findings are reassuring, though close and continuous monitoring should follow to allow continuous estimates.

The results of this study demonstrate the important implications of HIV-transmitted resistance mutations on viral fitness. K65R, in addition to conferring resistance to tenofovir, also leads to significant reductions in viral replication and fitness [15,16]. The consequence is a mutation that, according to our findings, is rarely detected even in settings with widespread use of tenofovir, which has been increasingly used as the main NRTI backbone of anti-retroviral regimens in developed countries in the past decade. Though uncommon and despite *in vitro* antagonism [17], K65R occurred with other mutations that impact viral fitness, including M184V in 35% (7/20) and L74V in 5% (1/20) of K65R-containing sequences.

Close surveillance is warranted as tenofovir-based regimens also become more widely used in developing countries for both HIV patient care as well as PrEP. Despite the reported paucity of K65R at the current time, a prevalence of 1% or greater in the HIV-positive population would still signify a large number of potentially transmissible viruses, especially in the setting of selective pressure from tenofovir.

The importance of quality control in sequence analyses should not be underestimated. Examination of sequences for this study yielded a significant number that had to be excluded, including sequences that did not encompass all the important resistance positions; sequences with many ambiguous nucleotides and stop codons suggesting low-quality sequencing; and duplicate sequences included in multiple studies. Rigorous pre-interpretation quality control of small and large sequence datasets included in analyses is essential.

The main limitation of this study is data availability. Our findings reflect the available sequences from reported studies, a similar limitation for each of the databases included here. Larger global datasets with more representative non-B subtypes are required to examine the occurrence of uncommon resistance mutations and their transmission. In addition, reliance on reported data is limited by its accuracy. We tried to overcome that by analyzing actual sequence data rather than relying on reported data in the papers, However, though reported from anti-retroviral-naïve patients, 7/20 K65R-containing sequences had three or more additional resistance mutations, raising the concern of prior drug exposure. In the context of this article, such circumstances would even lower the tenofovir-associated resistance transmission. Finally, the prevalence of reported K65R reviewed here may be underestimated due to the 15 to 20% detection threshold of population-based sequencing and the potential for DNA archiving [18].

## Conclusions

In summary, based on available literature and sequence data, the global transmission of K65R and other tenofovir-associated mutations is rare and should not affect tenofovir-based PrEP efficacy at this time. The acquisition of HIV prior to initiation of PrEP is a potential concern that may lead to drug resistance due to exposure to mono or dual anti-retroviral therapy. As tenofovir was FDA approved in 2001, the occurrence of transmitted and acquired related resistance may be delayed and therefore not yet reflected in reported data. Indeed, recent surveillance reports do indicate that K65R may be increasing in areas where non-B subtypes are predominant [19,20]. With global scale-up of anti-retroviral therapy, including tenofovir, continuous monitoring and reporting of transmitted drug resistance mutations will be essential to monitor impact on PrEP regimens.
